# The impact of COVID-19 infection on multiple sclerosis disease course across 12 countries: a propensity-score-matched cohort study

**DOI:** 10.1177/17562864241278496

**Published:** 2024-11-07

**Authors:** David Levitz, Yi Chao Foong, Paul Sanfilippo, Tim Spelman, Louise Rath, Angie Roldan, Anoushka Lal, Mastura Monif, Vilija Jokubaitis, Serkan Ozakbas, Raed Alroughani, Cavit Boz, Murat Terzi, Tomas Kalincik, Yolanda Blanco, Matteo Foschi, Andrea Surcinelli, Katherine Buzzard, Olga Skibina, Guy Laureys, Liesbeth Van Hijfte, Cristina Ramo-Tello, Aysun Soysal, Jose Luis Sanchez-Menoyo, Mario Habek, Elisabetta Cartechini, Juan Ignacio Rojas, Rana Karabudak, Barbara Willekens, Talal Al-Harbi, Yara Fragoso, Tamara Castillo-Triviño, Danny Decoo, Maria Cecilia Aragon de Vecino, Eli Skromne, Carmen-Adella Sirbu, Chao Zhu, Daniel Merlo, Melissa Gresle, Helmut Butzkueven, Anneke Van Der Walt

**Affiliations:** Department of Neuroscience, Central Clinical School, Monash University, Melbourne, VIC, Australia; Department of Neuroscience, Central Clinical School, Monash University, Melbourne, VIC, Australia; Department of Neurology, The Alfred Hospital, Melbourne, VIC, Australia; Department of Neuroscience, Central Clinical School, Monash University, Melbourne, VIC, Australia; Department of Clinical Neuroscience, Karolinska Institute, Stockholm, Sweden; Department of Neuroscience, Central Clinical School, Monash University, Melbourne, VIC, Australia; Department of Neuroscience, Central Clinical School, Monash University, Melbourne, VIC, Australia; Department of Neuroscience, Central Clinical School, Monash University, Melbourne, VIC, Australia; Department of Neuroscience, Central Clinical School, Monash University, Melbourne, VIC, Australia; Department of Neuroscience, Central Clinical School, Monash University, Melbourne, VIC, Australia; Dokuz Eylul University, Konak, Turkey; Division of Neurology, Department of Medicine, Amiri Hospital, Kuwait City, Kuwait; KTU Medical Faculty Farabi Hospital, Trabzon, Turkey; Medical Faculty, 19 Mayis University, Samsun, Turkey; Neuroimmunology Centre, Department of Neurology, The Royal Melbourne Hospital, Melbourne, VIC, Australia; CORe, Department of Medicine, University of Melbourne, Melbourne, VIC, Australia; Center of Neuroimmunology, Service of Neurology, Hospital Clinic de Barcelona, Barcelona, Spain; Department of Neuroscience, Neurology Unit, S. Maria delle Croci Hospital of Ravenna, AUSL Romagna, Ravenna, Italy; Department of Biotechnological and Applied Clinical Sciences (DISCAB), University of L’Aquila, L’Aquila, Italy; Department of Neuroscience, Neurology Unit, S. Maria delle Croci Hospital of Ravenna, AUSL Romagna, Ravenna, Italy; Department of Neurology, Box Hill Hospital, Melbourne, VIC, Australia; Eastern Health Clinical School, Monash University, Box Hill, VIC, Australia; Department of Neurology, The Alfred Hospital, Melbourne, VIC, Australia; Department of Neurology, Box Hill Hospital, Melbourne, VIC, Australia; Eastern Health Clinical School, Monash University, Box Hill, VIC, Australia; Department of Neurology, University Hospital Ghent, Ghent, Belgium; Department of Neurology, University Hospital Ghent, Ghent, Belgium; Hospital Germans Trias i Pujol, Badalona, Spain; Bakirkoy Education and Research Hospital for Psychiatric and Neurological Diseases, Istanbul, Turkey; Hospital de Galdakao-Usansolo, Galdakao, Spain; Instituto de Investigacion sanitario Biocruces-Bizkaia, Barakaldo, Spain; Department of Neurology, University Hospital Center Zagreb, Zagreb, Croatia; School of Medicine, University of Zagreb, Zagreb, Croatia; UOC Neurologia, Azienda Sanitaria Unica Regionale Marche – AV3, Macerata, Italy; Hospital Universitario de CEMIC, Buenos Aires, Argentina; Hacettepe University, Ankara, Turkey; Department of Neurology, Antwerp University Hospital, Edegem, Belgium; Translational Neurosciences Research Group, Faculty of Medicine and Health Sciences, University of Antwerp, Wilrijk, Belgium; King Fahad Specialist Hospital-Dammam, Dammam, Saudi Arabia; Universidade Metropolitana de Santos, Santos, Brazil; Hospital Universitario Donostia and IIS Biodonostia, San Sebastián, Spain; AZ Alma Ziekenhuis, Damme, Belgium; Hospital Moinhos de Vento, Porto Alegre, Brazil; Hospital Angeles de las Lomas, Instituto Mexicano de Neurociencias, Huixquilucan, Mexico; Central Military Emergency University Hospital, Bucharest, Romania; Titu Maiorescu University, Bucharest, Romania; Department of Neuroscience, Central Clinical School, Monash University, Melbourne, VIC, Australia; Department of Neurology, Box Hill Hospital, Melbourne, VIC, Australia; Department of Neuroscience, Central Clinical School, Monash University, Melbourne, VIC, Australia; Department of Neuroscience, Central Clinical School, Monash University, Melbourne, VIC, Australia; Department of Neurology, The Alfred Hospital, Melbourne, VIC, Australia; Department of Neuroscience, Central Clinical School, Monash University, 99 Commercial Road, Melbourne, VIC 3004, Australia; Department of Neurology, The Alfred Hospital, Melbourne, VIC, Australia

**Keywords:** COVID-19, disease progression, multiple sclerosis, relapse

## Abstract

**Background:**

The relationship between coronavirus disease 2019 (COVID-19) infection and multiple sclerosis (MS) relapse and disease progression remains unclear. Previous studies are limited by small sample sizes and most lack a propensity-matched control cohort.

**Objective:**

To evaluate the effect of COVID-19 infection on MS disease course with a large propensity-matched cohort.

**Design:**

This multi-centre cohort study analysed relapse and disability outcomes post-COVID-19 infection after balancing covariates using a propensity score matching method. The study period was from the 11th of September 2019 to the 16th of February 2023. The mean follow-up period was 1.7 years.

**Methods:**

Data were retrieved from the MSBase Registry. Propensity scores were obtained based on age, sex, disease duration, baseline Expanded Disability Status Scale (EDSS), MS course, relapses pre-baseline, disease-modifying therapy (DMT) class and country. Primary outcomes were time to first relapse, annualised relapse rate (ARR) and time to confirm EDSS progression. Secondary outcomes were time to EDSS of 3, 4 or 6. Sensitivity analyses with baseline DMT classes were performed.

**Results:**

The study included 2253 cases and 6441 controls. After matching, there were 2161 cases and an equal number of matched controls. Cases had a significantly higher ARR (ARR = 0.10 [95% CI 0.09–0.11]) compared to controls (ARR = 0.07 [95% CI 0.06–0.08]). Cases had a significantly greater hazard of time to first relapse compared to controls (hazard ratio (HR) = 1.54 [95% CI 1.29–1.84]). There was no association between COVID-19 infection and 24-week EDSS progression (HR = 1.18 [95% CI 0.92–1.52]), or time to EDSS of 3, 4 or 6. For patients on interferons and glatiramer acetate (BRACE), COVID-19 infection was associated with a greater hazard of time to first relapse (HR = 1.83 [95% CI 1.25–2.68]) and greater hazard of time to EDSS of 3 (HR = 2.04 [95% CI 1.06–3.90]) compared to patients on BRACE therapy without COVID-19 infection.

**Conclusion:**

COVID-19 infection was associated with a significantly increased MS relapse rate and a shorter time to first relapse. There was no effect on confirmed EDSS progression over the short term. These results support ongoing COVID-19 risk minimisation strategies to protect patients with MS.

## Background

Coronavirus disease 2019 (COVID-19) is an infectious disease caused by the severe acute respiratory syndrome coronavirus 2 (SARS-CoV-2). It was declared a public health emergency of international concern by the World Health Organization (WHO) in January 2020 and has caused nearly 7 million deaths worldwide.^1^

Previous studies have shown an association between viral infections, multiple sclerosis (MS) relapses and MRI lesions.^2–4^ While the exact mechanism is unclear, infections are thought to cause a systemic inflammatory response, leading to an increased expression of pro-inflammatory cytokines and chemokines. The alterations in the inflammatory mediators are crucial in the pathogenesis of COVID-19 symptoms and possibly could increase susceptibility to MS relapse.^5,6^ Given previous reports of viral infections as a trigger for demyelination, it is important to explore whether COVID-19 infection can lead to an increase in the rate of MS relapse or disability progression.^4^

The effect of COVID-19 on MS disease activity is unclear. Previous studies have shown inconsistent results likely due to their retrospective nature, small sample sizes and lack of propensity-matched controls.^7–15^ This study addresses these limitations by evaluating the effect of COVID-19 infection on MS disease course using a matched cohort study design.

## Methods

### Standard protocol approvals, registrations and patient consent

The MSBase Registry is an international observational cohort study of patients with MS (pwMS) established in 2000.^16^ The MSBase COVID-19 Substudy is an ongoing international and multi-site observational cohort substudy of MSBase comprising pwMS who have been infected (confirmed and suspected) with COVID-19. It was established in July 2020. Data for the analysis were extracted on the 16th of February 2023.

This study followed the Strengthening the Reporting of Observational Studies in Epidemiology (STROBE) reporting guidelines (Supplemental Material).

### Study inclusion criteria, data collection and definitions

Inclusion criteria for this study were as follows: (1) Definite diagnosis of MS; (2) Expanded Disability Status Scale (EDSS) baseline recorded within 6 months of the declaration of COVID-19 as a pandemic by the WHO (11 March 2020) for the control cohort^17^; (3) complete information for sex, age, duration of disease, the date of starting and/or stopping disease-modifying therapies (DMTs), EDSS assessments and dates of relapses for the duration of the study; (4) at least two follow-up visits with a minimum 6-month gap and complete EDSS assessments to allow for the calculation of confirmed disability progression (CDP); and (5) 18 years of age or over. Exclusion criteria for this study were diagnosis of neuromyelitis optica spectrum disorder (NMOSD), myelin oligodendrocyte glycoprotein antibody disorder (MOGAD) or radiologically isolated syndrome (RIS). All MSBase members were invited to participate in this study, with 25 centres accepting the invitation. We included pwMS diagnosed with COVID-19 on polymerase chain reaction (PCR) or rapid antigen (RAT) tests. Cases were included from the time of COVID-19 infection to the time of their last recorded registry visit if they met inclusion criteria. Patients from the participating MSBase COVID-19 Substudy centres meeting the above criteria but without documented COVID-19 infection were used as a control cohort.

These medications – natalizumab, ocrelizumab, rituximab, alemtuzumab, cladribine, fingolimod, siponimod and ofatumumab – were classified as high-efficacy DMTs. All others were classified as low-moderate efficacy DMTs.

DMT classes described for each patient were recorded at baseline.

Patient data were recorded during routine clinical visits at participating centres via the locally installed iMed or MSBase data entry systems (MDS).

Baseline EDSS for the control cohort was defined as the closest visit within 6 months of the declaration of COVID-19 as a pandemic by the WHO (11 March 2020). Cases were included from the date of their infection and subsequently followed up.

### Study endpoints

The primary study outcomes were annualised relapse rate (ARR, calculated by dividing the total number of relapses by the total number of person-years at risk), time to first relapse and 24-week CDP.

CDP was defined as an increase of ⩾1.5 EDSS steps from a baseline score of 0, 1 step from baseline scores 1.0–5.5 or 0.5 step from a baseline score ⩾5.5, sustained at two or more consecutive visits separated by ⩾180 days.^18^

Relapse was defined as a clinical episode lasting at least 24 h, in the absence of fever, infection or acute concurrent medical illness. There also must have been a preceding 30-day period without clinical relapse.

Secondary outcomes were time to EDSS of 3, 4 or 6. We also repeated the primary and secondary outcomes stratified by baseline DMT classes. DMTs were categorised as glatiramer acetate/interferon beta-1 (BRACE), teriflunomide, dimethyl fumarate, sphingosine-1-phosphate receptor antagonists (fingolimod, siponimod, ponesimod, ozanimod), anti-CD20s (ocrelizumab, ofatumumab, rituximab) and other monoclonal antibodies (natalizumab, alemtuzumab).

### Statistical analysis

The demographic information and the baseline characteristics were reported as numbers and percentages for discrete variables and as mean (standard deviation (SD)) or median (interquartile range (IQR)) for continuous variables, as appropriate.

We applied 1:1 propensity score matching to match COVID-19 cases and controls to mitigate baseline differences between the groups and selection bias.^19^ The matching method used was k-nearest neighbour matching with a caliper of 0.05. The propensity score was calculated using logistic regression with COVID-19 infection as the dependent outcome variable and the following baseline predictors as explanatory covariates: age, sex, disease duration, baseline EDSS, MS course, relapses pre-baseline, DMT class and country. The covariate balance was evaluated using the absolute standardised difference (ASD) measure, where ASD > 0.1 indicates an imbalance.^20^

A negative binomial model was used to compare ARRs, with the relapse count as a dependent variable, and COVID-19 as an independent variable, offset by the follow-up time. We used marginal Cox regression models with robust standard errors and Kaplan–Meier cumulative hazard curves to assess and visualise differences between cases and controls for the time-to-event outcomes. Participants were censored at the time of the event or the last available follow-up visit in those without an event.

We then ran a sensitivity analysis for the relapsing–remitting multiple sclerosis (RRMS) subgroup to assess the potential impact of confounding errors that may result from the inclusion of different MS subtypes.

All statistical tests were two-sided with a statistical significance defined as *p* < 0.05. All analyses were performed in R, V.4.1.1 (R Foundation for Statistical Computing, Vienna, Austria).

## Results

### Participants

**Figure 1.**
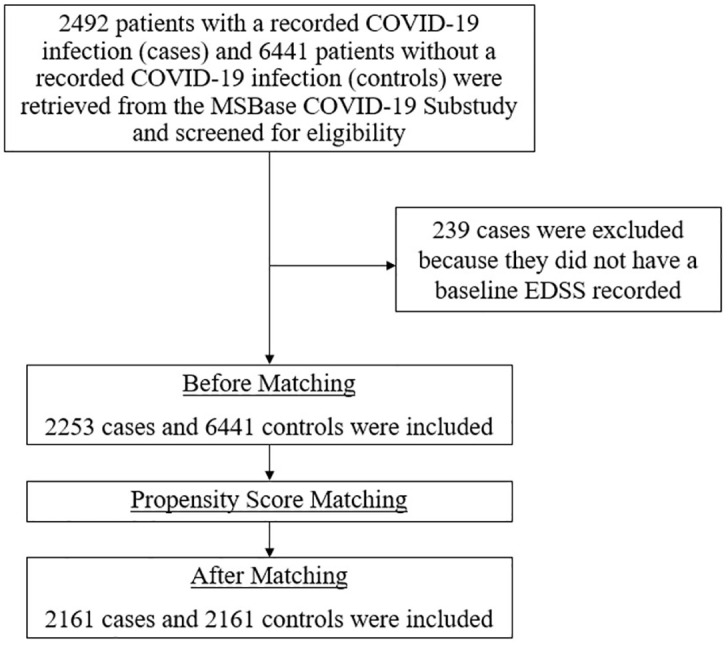
Flow diagram of patients selected from the MSBase COVID-19 registry and 1:1 propensity score matching of cases and controls. COVID-19, coronavirus disease 2019; EDSS, Expanded Disability Status Scale.

A total of 2253 cases and 6441 controls were included (baseline characteristics in Table A1). At baseline, COVID-19 cases were younger, had shorter disease duration, had lower baseline EDSS, had lower pre-baseline relapse activity and were more likely to be on high-efficacy DMTs. After propensity score matching, 2161 cases and an equal number of controls were identified. Baseline characteristics of the matched study population are presented in Table 1. After matching, all baseline covariates were balanced (ASD < 0.1). In all, 239 patients infected with COVID-19 were excluded because they had no baseline EDSS recorded (Figure 1). The distribution of COVID-19 severity in the cohort was: 2.64% had severe COVID-19 infection (ICU admission), 10.00% had moderate severity COVID-19 infection (inpatient admission), 80.66% had mild COVID-19 infection (outpatient setting) and 6.71% had no severity reported.

**Table 1.** Baseline characteristics of the matched study population by COVID cases versus controls.

Baseline factor	Category/metric	Cases (*n* = 2161)	Controls (*n* = 2161)	Standardised difference
Age	Mean (SD)	40.63 (11.41)	40.45 (11.68)	0.02
Sex	Female	1506 (69.7)	1515 (70.1)	−0.01
	Male	655 (30.3)	646 (29.9)	
Disease duration (years)	Mean (SD)	9.71 (8.18)	9.94 (7.23)	−0.03
Baseline EDSS	Median (IQR)	1.5 (1–3)	1.5 (1–3)	0.02
MS course	CIS	177 (8.2)	154 (7.1)	0.03
	PP	76 (3.5)	139 (6.4)	
	PR	24 (1.1)	21 (1.0)	
	RR	1751 (81.0)	1733 (80.2)	
	SP	133 (6.2)	96 (4.4)	
	Not reported	0 (0.0)	18 (0.8)	
Relapses 1-year pre-baseline	Mean (SD)	0.07 (0.30)	0.07 (0.29)	−0.02
Relapses 2-year pre-baseline	Mean (SD)	0.10 (0.43)	0.11 (0.39)	−0.03
DMT class	High efficacy	1058 (49.0)	1109 (51.3)	0.03
	Low-moderate efficacy	746 (34.5)	686 (31.7)	
	No baseline DMT	357 (16.5)	366 (16.9)	
Country	Argentina	20 (0.9)	67 (3.1)	−0.03
	Australia	254 (11.8)	271 (12.5)	
	Belgium	52 (2.4)	109 (5.0)	
	Brazil	21 (1.0)	19 (0.9)	
	Spain	231 (10.7)	177 (8.2)	
	Croatia	179 (8.3)	7 (0.3)	
	Italy	285 (13.2)	93 (4.3)	
	Kuwait	137 (6.3)	363 (16.8)	
	Mexico	3 (0.1)	2 (0.1)	
	Romania	3 (0.1)	2 (0.1)	
	Saudi Arabia	9 (0.4)	27 (1.3)	
	Turkey	967 (44.8)	1024 (47.4)	

Values are expressed as *n* (%), median (IQR) or mean (SD). Standardised difference is the difference in means or proportions divided by the standard error. An imbalance was defined as an ASD > 0.10.

CIS, clinically isolated syndrome; DMT, disease-modifying therapy; EDSS, Expanded Disability Status Scale; IQR, interquartile range; MS, multiple sclerosis; PP, primary progressive; PR, progressive relapsing; RR, relapsing remitting; SD, standard deviation; SP, secondary progressive.

### Primary analyses

**Figure 2.**
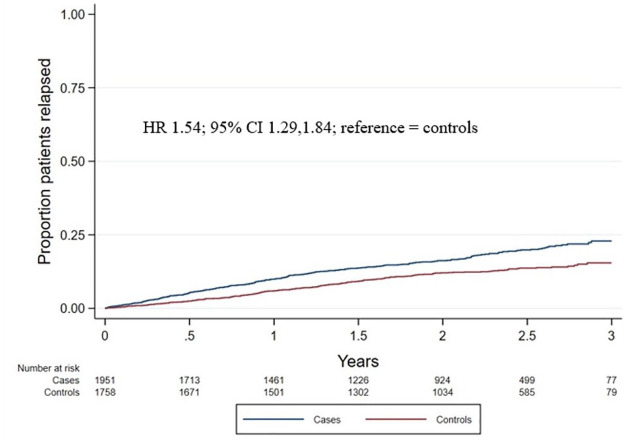
Cumulative hazard of time to first relapse. Kaplan–Meier curves were applied to show the cumulative hazard of time to first relapse in COVID-19 cases (blue line) and controls (red line).

The total number of post-baseline relapses was 380 in COVID-19 cases and 255 in controls. Follow-up person-years were 3689 and 3718 for COVID-19 cases and controls, respectively. COVID-19 infection in pwMS was associated with a significantly higher ARR (ARR = 0.10 [95% CI 0.09–0.11]) compared to matched controls without COVID-19 infection (ARR = 0.07 [95% CI 0.06–0.08]). Consistent results were found for time to first relapse, with cases having a significantly greater hazard of time to first relapse compared to controls (hazard ratio (HR) = 1.54 [1.29, 1.84]; Figure 2).

Sensitivity analysis of the RRMS subgroup demonstrated no significantly different results. COVID-19 infection in pwMS with the RRMS subtype was associated with a significantly higher ARR (ARR = 0.1009 [0.0901, 0.1126]) compared to matched controls without COVID-19 infection (ARR = 0.0735 [0.0642, 0.0838]). Consistent results were found for time to first relapse, with cases having a significantly greater hazard of time to first relapse compared to controls (HR = 1.40 [1.16, 1.69]).

**Figure 3.**
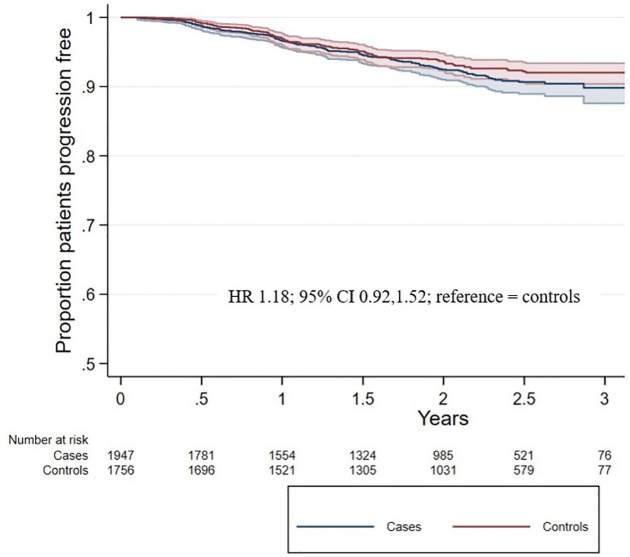
Cumulative hazard of time to CDP. Kaplan–Meier curves were applied to show the cumulative hazard of time to first relapse in COVID-19 cases (blue line) and controls (red line). CDP, confirmed disability progression.

Of the COVID-19 cases, 133 (6.2%) experienced a CDP event, while 111 (5.1%) controls experienced a CDP event. There was no association between COVID-19 infection and 24-week EDSS progression (HR = 1.18 [0.92, 1.52]; Figure 3). The sensitivity analysis for the RRMS subtype also found no association between COVID-19 infection and 24-week EDSS progression (HR = 1.19 [0.86, 1.65]).

### Secondary analyses

**Figure A1.**
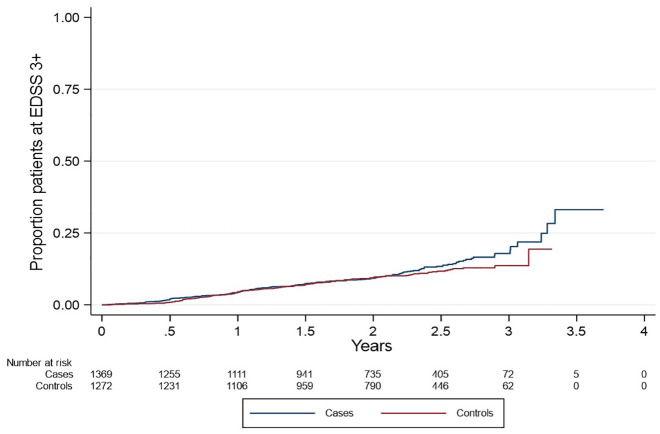
Cumulative hazard of time to EDSS equal or greater than 3. Weighted Kaplan–Meier curves were applied to show the cumulative hazard of time to first relapse in COVID-19 cases (blue line) and controls (red line). EDSS, Expanded Disability Status Scale.

**Figure A2.**
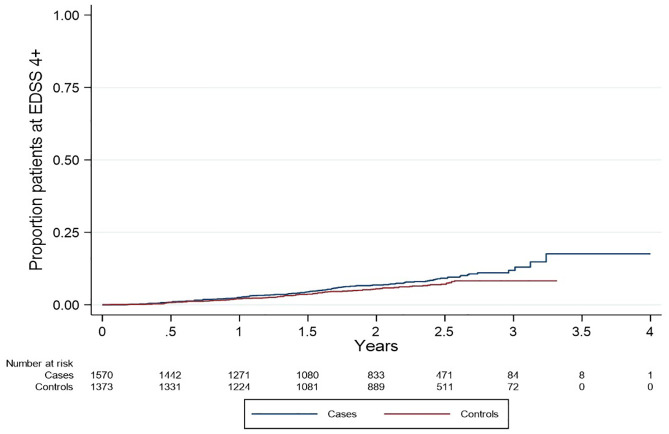
Cumulative hazard of time to EDSS equal or greater than 4. Weighted Kaplan–Meier curves were applied to show the cumulative hazard of time to first relapse in COVID-19 cases (blue line) and controls (red line). EDSS, Expanded Disability Status Scale.

**Figure A3.**
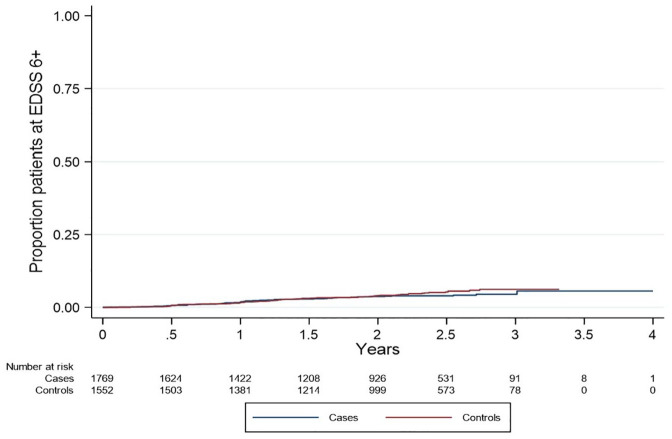
Cumulative hazard of time to EDSS equal or greater than 6. Weighted Kaplan–Meier curves were applied to show the cumulative hazard of time to first relapse in COVID-19 cases (blue line) and controls (red line). EDSS, Expanded Disability Status Scale.

There was no association between COVID-19 infection and time to EDSS of 3 (HR = 1.17 [0.93, 1.49]), 4 (HR = 1.31 [0.99, 1.74]) or 6 (HR = 0.84 [0.59, 1.19]; Figures A1–A3, respectively).

COVID-19 cases who were on interferons or glatiramer acetate (BRACE) had a greater hazard of time to first relapse (HR = 1.54 (95% CI 1.29–1.84)) and time to EDSS of 3 (HR = 2.04 (95% CI 1.06–3.90)) compared to similarly treated controls. There was no difference in the hazard of time to first relapse, CDP or time to EDSS or 3, 4 or 6 for other intra-DMT comparisons (Table 2).

**Table 2.** Hazard ratios of COVID-19 cases versus controls for time to first relapse, confirmed disability progression and time to EDSS of 3, 4 and 6, stratified by DMT classes.

Outcomes	Overall (*n* = 4322)	BRACE (*n* = 733)	Teriflunomide (*n* = 267)	DMF (*n* = 354)	S1PR modulators (*n* = 887)	Anti-CD20s (*n* = 882)	Other mAbs (*n* = 389)	Cladribine (*n* = 73)
	Hazard ratio (95% CI) *p* value*	Hazard ratio (95% CI) *p* value*	Hazard ratio (95% CI) *p* value*	Hazard ratio (95% CI) *p* value*	Hazard ratio (95% CI) *p* value*	Hazard ratio (95% CI) *p* value*	Hazard ratio (95% CI) *p* value*	Hazard ratio (95% CI) *p* value*
Time to first relapse	**1.54 (1.29, 1.84) <0.001**	**1.83 (1.25, 2.68) 0.002**	1.60 (0.87, 2.95) 0.134	1.71 (0.92, 3.19) 0.092	1.25 (0.86, 1.84) 0.246	1.50 (0.89, 2.54) 0.132	1.74 (0.86, 3.52) 0.126	2.16 (0.43, 10.70) 0.347
24-week CDP	1.18 (0.92, 1.52) 0.198	1.02 (0.36, 2.93) 0.963	0.98 (0.34, 2.80) 0.975	1.25 (0.44, 3.54) 0.679	1.13 (0.58, 2.20) 0.710	0.86 (0.57, 1.30) 0.466	1.99 (0.78, 5.04) 0.149	0.72 (0.10, 5.14) 0.747
Time to EDSS ⩾ 3	1.17 (0.93, 1.49) 0.181	**2.04 (1.06, 3.90) 0.032**	0.53 (0.21, 1.33) 0.177	0.81 (0.37, 1.77) 0.596	1.13 (0.65, 1.94) 0.671	0.79 (0.47, 1.35) 0.390	1.73 (0.85, 3.50) 0.129	0.78 (0.15, 3.94) 0.761
Time to EDSS ⩾ 4	1.31 (0.99, 1.74) 0.062	1.85 (0.78, 4.43) 0.164	0.90 (0.32, 2.48) 0.834	0.75 (0.27, 2.10) 0.581	1.93 (0.96, 3.88) 0.065	0.90 (0.50, 1.60) 0.712	0.83 (0.35, 1.97) 0.677	0.37 (0.03, 4.27) 0.426
Time to EDSS ⩾ 6	0.84 (0.59, 1.19) 0.321	1.66 (0.40, 6.95) 0.488	0.34 (0.04, 3.25) 0.347	0.58 (0.04, 9.20) 0.696	1.67 (0.53, 5.26) 0.381	0.63 (0.38, 1.04) 0.072	0.72 (0.24, 2.22) 0.572	1.28 (0.12, 14.18) 0.841

Values are expressed as hazard ratio (95% CI) *p* value. Controls (pwMS without COVID-19) were taken to be the reference group in the above analyses. Bold values indicate statistical significance at *p* < 0.05.

BRACE, glatiramer acetate/interferon beta-1; CDP, confirmed disability progression; DMF, dimethyl fumarate; DMT, disease-modifying therapy; EDSS, Expanded Disability Status Scale; mAbs, monoclonal antibodies; MS, multiple sclerosis; S1PR, sphingosine-1-phosphate receptor; SD, standard deviation

## Discussion

In this large multi-national observational cohort study, we demonstrate that pwMS infected with COVID-19 have a significantly increased relapse rate and shorter time to first relapse than matched controls without COVID-19 infection. However, there is no difference in confirmed EDSS progression between the groups. This suggests that COVID-19 infection can acutely exacerbate neurological symptoms. Our results suggest that COVID-19 potentially does not increase disability progression over 1.7 years of follow-up. However, these analyses need to be repeated with longer follow-up times in the future.

The association of COVID-19 infection and higher ARR is consistent with previous reports of viral infections as a trigger for demyelination and clinical relapse in pwMS.^2–4^ Reports of other respiratory tract infections leading to an increased risk of relapse in pwMS are also consistent with our findings.^2,3^ Many pathophysiological processes have been postulated to explain this process. Preliminary studies looking at the effect of COVID-19 on the immune system have suggested COVID-19 infection is characterised by misdirected host immune responses that can exacerbate pre-existing autoimmune disease.^21^ Furthermore, the increased pro-inflammatory state caused by COVID-19 infection may lead to the recruitment and activation of pro-inflammatory leucocytes ‘bystanders’ that can cross the blood–brain barrier, increasing the risk of clinical MS relapse.^6^ The literature on the relationship between COVID-19 and MS relapse has been controversial. For example, studies by Etemadifar et al.^13^ and Bsteh et al.^22^ found no association between COVID-19 and relapse, or even a lower exacerbation incidence in pwMS infected with COVID-19. However, these studies were limited by small sample sizes.

Including a time variable in our analysis allows us to evaluate the effect of COVID-19 on the time to first relapse. Other studies have used a binary relapse variable to assess the risk of relapse by comparing MS exacerbation in a cohort for a fixed time before the pandemic and a fixed time before and after COVID-19 infection.^12,13^

Our study found no association between COVID-19 and EDSS progression. This is consistent with several smaller studies. However, these results contradict findings from Peeters et al.,^7^ who suggest that COVID-19 is linked to clinical worsening in MS patients. This study had a relatively small sample size and no control group. A systematic review and meta-analysis by Aghajanian et al.^23^ indicated no impact of COVID-19 infection on MS relapse rates. However, the studies in this review had small sample sizes, limited follow-up duration and did not evaluate incidence through time-to-event analyses. Similarly, a propensity score-matched case-control study by Vercellino et al.^24^ found no differences in relapse or MRI activity post-COVID-19 infection, though it also did not analyse relapse incidence through time-to-event methods.^24^

Our study also looked at the effect of DMTs on outcome measures. Patients with COVID-19 on BRACE therapies demonstrated a reduced time to first relapse and time to EDSS of 3. Low-efficacy medications are not as effective as other DMTs in suppressing MS activity. As a result, a viral infection like COVID-19 could exacerbate partially controlled MS-related inflammatory activity and result in a relapse. However, these findings must be interpreted with caution, as other unmeasured confounding factors could influence DMT choice and relapse activity in this cohort. For example, insurance status, variation in prescribing at a site level, smoking and obesity were not measured. The BRACE analysis findings should be understood in the context of reports that COVID-19 infection may be less severe in pwMS on BRACE therapy.^25^ Our study suggests that the benefit of less severe COVID-19 infection in these patients must be balanced against the increased possibility of MS relapse triggered by COVID-19.

A substantial body of literature suggests that anti-CD20 therapies can worsen the severity of COVID-19.^26–31^ However, other studies suggest that ocrelizumab is not a risk factor for severe COVID-19 infection, indicating the need for further research.^32^ We did not find an association between COVID-19 infection and MS disease course for pwMS on anti-CD20 therapies. This may suggest that anti-CD20 therapies effectively suppress MS disease activity, limiting infection-related relapse activity. Again, this calls for a balance between minimising the severity of COVID-19 infection and mitigating MS disease progression and relapse.

These results support COVID-19 risk minimisation strategies to protect pwMS from neurological exacerbation and relapse. It is also important that pwMS are aware of the risk of infection with COVID-19 on MS relapse and neurological exacerbation to make informed decisions about their lifestyle and health. These decisions should also consider the specific DMT therapy the patient is on and include other disease-related factors such as age, sex, disease duration, MS course and history of past relapse. While the WHO has declared that COVID-19 is no longer a global health emergency, it remains important for pwMS and healthcare providers to be vigilant about the continued presence of this disease in the community.^33^

### Strengths and limitations

The strengths of this study include the analysis of data from 25 sites incorporating populations from 12 countries. Including multiple sites increases the generalisability of these findings and the large sample size reduces selection bias and improves the accuracy of results. The propensity-matched control cohort mitigated confounding factors such as age, sex, history of relapse, disease duration, baseline EDSS, MS course, DMT class (high efficacy, low-moderate efficacy, no baseline DMT) and country.^34–39^ Including a time variable allowed for the calculation of the hazard ratio, which is a unique outcome variable of this paper.

There are some limitations to our study. A lack of PCR sequencing means that results cannot be stratified to different strains of COVID-19. Inconsistent vaccine data across sites meant we could not assess the effect of COVID-19 vaccination on relapse and disability. Although we were unable to incorporate vaccination data in our analysis, it is important to address potential interactions between vaccination and our outcomes of interest. This retrospective, observational cohort study found a mild increase in ARR after vaccination.^40^ However, more recent studies have not found increased relapse activity after COVID-19 vaccine administration.^41^ A systematic review and meta-analysis by Stefanou et al.^42^ included 19 observational studies consisting of 14,755 pwMS and concluded that COVID-19 vaccination did not appear to increase the risk of relapse. A limitation of this review is that it only consists of observation studies and so results are only single-arm estimates due to the lack of robust control cohorts.

Additional potential confounders that may affect outcomes post-COVID-19, such as comorbidities, frailty and body composition, were not controlled for in our study.^38,43–46^ Confounders described to be associated with an increased risk of hospitalisation in pwMS and COVID-19 such as diabetes, smoking history and hypertension were not accounted for in our analysis.^47^

There is a lack of Multiple Sclerosis Functional Composite score which could indicate the possibility of progression independent of relapse activity. We could not corroborate relapse with MRI findings. It is possible that some members of the MSBase control cohort, who were not reported to have COVID-19, may have had an unknown or unreported past infection. To minimise the risk of selecting patients with unknown COVID-19 infection, controls were retrieved from sites participating in the MSBase COVID-19 Substudy. Additionally, there may be a reporting bias of COVID-19 in patients presenting with relapse. We conducted a sensitivity analysis for the RRMS subgroup to assess the potential impact of confounding errors that may result from the inclusion of different MS subtypes. This demonstrated no significant difference in results compared to the total cohort. We have also not included the severity of COVID-19, so we cannot comment on its relationship with the MS disease course. The severity and duration of relapse were not represented in our study. Some countries had different quarantine measures based on age. This may have impacted exposure to COVID-19 infection, as well as access to MS services or pharmacies to receive usual (prescribed) MS DMTs. This study only analysed the effect of baseline DMTs. It did not consider the duration or proportion of time a patient was on specific DMT therapies. The interpretation of the outcome measure of CDP may also be limited in this paper given the relatively short follow-up period of 1.7 years. The cessation of DMTs and irregular compliance were not accounted for in our analysis. If treatment was interrupted due to infection, reported increased relapse rates may be confounded by MS disease activity rather than be caused by COVID-19 directly.

## Conclusion

In our study, COVID-19 infection was associated with a significantly increased MS relapse rate and a shorter time to first relapse. There was no difference in confirmed EDSS progression over a short follow-up period of 1.7 years. BRACE therapy was associated with a greater hazard of time to first relapse and time to EDSS of 3 in cases compared to controls.

## Supplemental Material

sj-docx-1-tan-10.1177_17562864241278496 – Supplemental material for The impact of COVID-19 infection on multiple sclerosis disease course across 12 countries: a propensity-score-matched cohort study Supplemental material, sj-docx-1-tan-10.1177_17562864241278496 for The impact of COVID-19 infection on multiple sclerosis disease course across 12 countries: a propensity-score-matched cohort study by David Levitz, Yi Chao Foong, Paul Sanfilippo, Tim Spelman, Louise Rath, Angie Roldan, Anoushka Lal, Mastura Monif, Vilija Jokubaitis, Serkan Ozakbas, Raed Alroughani, Cavit Boz, Murat Terzi, Tomas Kalincik, Yolanda Blanco, Matteo Foschi, Andrea Surcinelli, Katherine Buzzard, Olga Skibina, Guy Laureys, Liesbeth Van Hijfte, Cristina Ramo-Tello, Aysun Soysal, Jose Luis Sanchez-Menoyo, Mario Habek, Elisabetta Cartechini, Juan Ignacio Rojas, Rana Karabudak, Barbara Willekens, Talal Al-Harbi, Yara Fragoso, Tamara Castillo-Triviño, Danny Decoo, Maria Cecilia Aragon de Vecino, Eli Skromne, Carmen-Adella Sirbu, Chao Zhu, Daniel Merlo, Melissa Gresle, Helmut Butzkueven and Anneke Van Der Walt in Therapeutic Advances in Neurological Disorders

## Appendix

**Table A1.** Baseline characteristics of unmatched study population by COVID cases versus controls.

Baseline factor	Category/metric	Cases (*n* = 2253*)	Controls (*n* = 6441)	Standardised difference
Age	Mean (SD)	40.26 (11.45)	43.03 (12.59)	−**0.230**
Sex	Female	1576 (70.0)	4474 (69.5)	0.011
	Male	677 (30.0)	1967 (30.5)	
Disease duration (years)	Mean (SD)	9.45 (8.14)	11.34 (8.99)	−**0.221**
Baseline EDSS	Median (IQR)	1.5 (1, 3)	2 (1, 4)	−**0.130**
MS course	CIS	254 (11.3)	301 (4.7)	−**0.257**
	PP	85 (3.8)	292 (4.5)	
	PR	24 (1.1)	70 (1.1)	
	RR	1757 (78.0)	5109 (79.3)	
	SP	133 (5.9)	530 (8.2)	
	Not reported	0 (0.0)	139 (2.2)	
Relapses 1-year pre-baseline	Mean (SD)	0.06 (0.29)	0.21 (0.50)	−**0.353**
Relapses 2-year pre-baseline	Mean (SD)	0.10 (0.42)	0.39 (0.72)	−**0.496**
DMT class	High efficacy	1125 (49.9)	2403 (37.3)	−**0.331**
	Low-moderate efficacy	771 (34.2)	2182 (33.9)	
	No baseline DMT	357 (15.9)	1856 (28.8)	
Country	Argentina	20 (0.9)	158 (2.5)	−**0.257**
	Australia	265 (11.8)	984 (15.3)	
	Belgium	52 (2.3)	364 (5.7)	
	Brazil	21 (0.9)	65 (1.0)	
	Spain	231 (10.3)	865 (13.4)	
	Croatia	214 (9.5)	24 (0.4)	
	Italy	296 (13.1)	392 (6.1)	
	Kuwait	149 (6.6)	821 (12.8)	
	Mexico	3 (0.1)	8 (0.1)	
	Romania	3 (0.1)	2 (0.0)	
	Saudi Arabia	9 (0.4)	66 (1.0)	
	Turkey	990 (43.9)	2692 (41.8)	

Values are expressed as *n* (%), median (IQR) or mean (SD). Standardised difference is the difference in means or proportions divided by the standard error. An imbalance was defined as an ASD > 0.10, indicated in bold.

* 239 of the original 2492 cases were excluded for not having a baseline EDSS

ASD, absolute standardised difference; CIS, clinically isolated syndrome; DMT, disease-modifying therapy; EDSS, Expanded Disability Status Scale; MS, multiple sclerosis; PP, primary progressive; PR, progressive relapsing; RR, relapsing remitting; SP, secondary progressive; SD, standard deviation.

## Declarations

**Ethics approval and consent to participate:** Ethics approval for the collection of demographics, COVID-19, clinical and treatment data via the MSBase Registry was obtained from the Alfred Health Human Research Ethics Committee (528/12), and institutional review boards at all participating centres. Written informed consent was obtained from all enrolled registry participants.

**Consent for publication:** Not applicable.

**Availability of data and materials:** *Data: Data available: Yes.* Data types: Deidentified participant data, Data dictionary. How to access data: https://www.msbase.org/media/1197/mds-datadictionary.pdf. When available: With publication. *Supporting Documents*: Document types: None. *Additional Information*: Who can access the data: Researchers whose proposed use of the data has been approved. *Types of analyses*: For repeating the analysis. *Mechanisms of data availability*: Permission from each contributing data controller is required.
